# Foot drop after spinal anaesthesia: A rare complication

**DOI:** 10.4103/0019-5049.76590

**Published:** 2011

**Authors:** BC Nirmala, Gowri Kumari

**Affiliations:** Department of Anaesthesia, MVJ Medical College and Research Hospital, Hoskote, Bangalore, Karnataka, India

Sir,

We report a case of foot drop following spinal anaesthesia. As such, neurological complications for central neuraxial blocks are less common. At the time of spinal anaesthesia, if the patient complains of pain or paraesthesia, watch these cases postoperatively carefully for any neurological deficit. The most likely cause in our case is neurotrauma.

A healthy 28-year-old adult female was scheduled for diagnostic laparoscopy. She had no other medical comorbidity history. After obtaining informed written consent and after overnight fasting, she was pre-medicated with tab lorazepam 2 mg, tab ranitidine 150 mg and tab ondensetron 4 mg at 6 AM on the day of surgery (3 h before shifting to the operating room).

In the operative room, an intravenous (IV) access was obtained with a 18 g cannula and monitoring with electrocardiogram (ECG), pulse oximetry and non invasive blood pressure (NIBP) were initiated. With all aseptic precautions a 25g Quincke needle was inserted in the L3-L4 interspace. As the needle entered the subarachnoid space, the patient presented a jerky reaction and complained of paraesthesia and pain. Immediately, the needle was withdrawn, following which the pain subsided instantaneously and she was comfortable. A second time, the needle was placed in the L3-L2 interspace uneventfully. There was good cerebrospinal fluid flow and 3 ml of 0.5% bupivacaine with 25 μg of fentanyl[1] were injected. Inj midazolam 1 mg was given intravenously. The laparoscopic procedure lasted for 35 min. Intraoperative hypotension was managed with Inj ephedrine and her haemodynamic values were maintained stable. The patient was lightly sedated and comfortable throughout.

Postoperatively, approximately after 4 h, the patient was ambulated for passing urine. Then, she noticed that she was unable to move her right foot and the left foot was normal. On examination, she had weakness of the right foot. The case was referred to the neurology department. They diagnosed right-sided foot drop (4/5) and started her on steroids nonsteroidal anti-inflammatory drugs (NSAID) and B-complex. Neurological examination was performed every 4th hourly[2,3] to observe for any progressive weakness. The patient’s neurological symptoms were nonprogressive. On the following day, magnetic resonance imaging was performed, which showed no abnormality [Figure 1]. She was discharged with advice of physiotherapy and to continue steroids NSAID and B-complex on the 5^th^ day.

**Figure 1:**
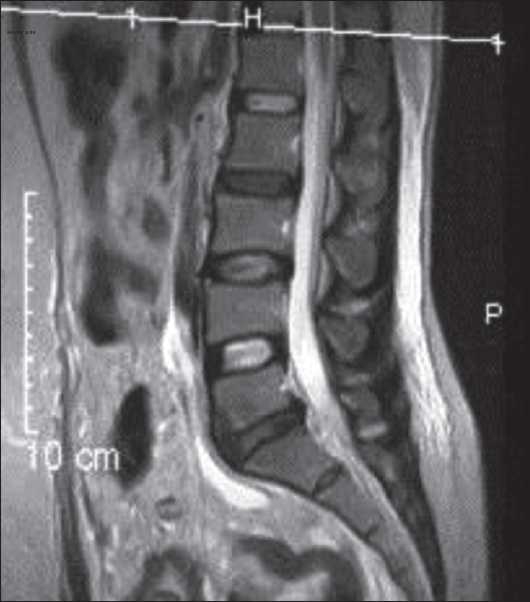
Normal magnetic resonance imaging

In the follow-up after 4 weeks, she had partial recovery of (3/5) motor power and 8 weeks later, she had complete recovery.

Spinal anaesthesia with opioids provides excellent surgical conditions for short laparoscopic surgeries.[4] During neuraxial blockade, trauma to the nerve roots or spinal cord could be the cause for paraesthesia and pain. Needle trauma or accidental wrong drug placement are the probable causes for neurological complications following spinal anaesthesia.

Nerve conduction studies are useful in the localization and assessment of nerve injury. Electromyography studies are an adjunct to nerve conduction studies. Signs of denervation on the electromyogram (EMG) after acute nerve injury require 18–21 days to develop.

However, the type and extent of nerve injury vary with the orientation of the needle. When the bevel is parallel to the long axis of the nerve, the needle more readily pass between the fibres. When the needle is transverse to the nerve fibre, the injury is greater.[5] Some sensory disturbances and occasional weakness may last for more than a year.

When paraesthesia is elicited as the needle advances, one can be reasonably certain that a nerve root has been struck. Paraesthesia and its intensity may serve as a warning sign and a guide for severity of nerve injury. Never inject any drug when the patient complains of pain, as an intraneural injection is painful and forebodes permanent damage.

## References

[1] Hamber EA, Viscomi CM (1999). Intrathecal lipophlic opioids as adjuncts to surgical spinal anaesthesia. Reg Anesth Pain Med.

[2] Reynold’s F (2001). Damage to the conus medullaris following spinal anaesthesia. Anaesthesia.

[3] Auroy Y, Narchi P, Messiah A, Litt L, Rouvier B, Samii K (1997). Serious complications related to regional anaesthesia: Results of a prospective survey in France. Anaesthesiology.

[4] de Santiago J, Santos-Yglesias J, Giron J, Montes de Oca F, Jimenez A, Diaz P (2009). Low dose 3mg levobupivacaine plus 10μg fentanyl selective spinal anaesthesia for gynecological outpatient laparoscopy. Anesth Analg.

[5] Selander D, Dhuner KG, Lundborg G (1997). Peripheral nerve injury due to injection needles used for regional anaesthesia. An experimental study of the acute effects of needle point trauma. Acta Anaesthesiol Scand.

